# Supplementary material for: Role of Liver Kinase 1B in Platelet Activation and Host Defense During Klebsiella pneumoniae-Induced Pneumosepsis
Source: Int J Mol Sci. 2025 Apr 14;26(8):3714. doi: 10.3390/ijms26083714 (PMC12028316; doi:10.3390/ijms26083714)
Supplement: Supplementary file 1 [file ijms-26-03714-s001.zip › ijms-3456473-supplementary.pdf]

Supplementary Materials

Role of Liver Kinase 1B in Platelet Activation and Host Defense During *Klebsiella pneumoniae*-Induced Pneumosepsis

Osoul Chouchane [1], Valentine Léopold [1], Christine C. A. van Linge [1], Alex F. de Vos [1], Joris J. T. H. Roelofs [2], Cornelis van 't Veer [1] and Tom van der Poll [1,3,*]

[1] Center of Infection and Molecular Medicine, Amsterdam University Medical Center, Location Academic Medical Center, University of Amsterdam, 1105AZ Amsterdam, The Netherlands; o.chouchane@amsterdamumc.nl (O.C.); v.leopold@amsterdamumc.nl (V.L.); c.c.vanlinge@amsterdamumc.nl (C.C.A.v.L.); a.f.devos@amsterdamumc.nl (A.F.d.V.); c.vantveer@amsterdamumc.nl (C.v.'t.V.)

[2] Department of Pathology, Amsterdam University Medical Center, Location Academic Medical Center, University of Amsterdam, 1105AZ Amsterdam, The Netherlands; j.j.roelofs@amsterdamumc.nl

[3] Division of Infectious Diseases, Amsterdam University Medical Center, Location Academic Medical Center, University of Amsterdam, 1105AZ Amsterdam, The Netherlands

* Correspondence: t.vanderpoll@amsterdamumc.nl

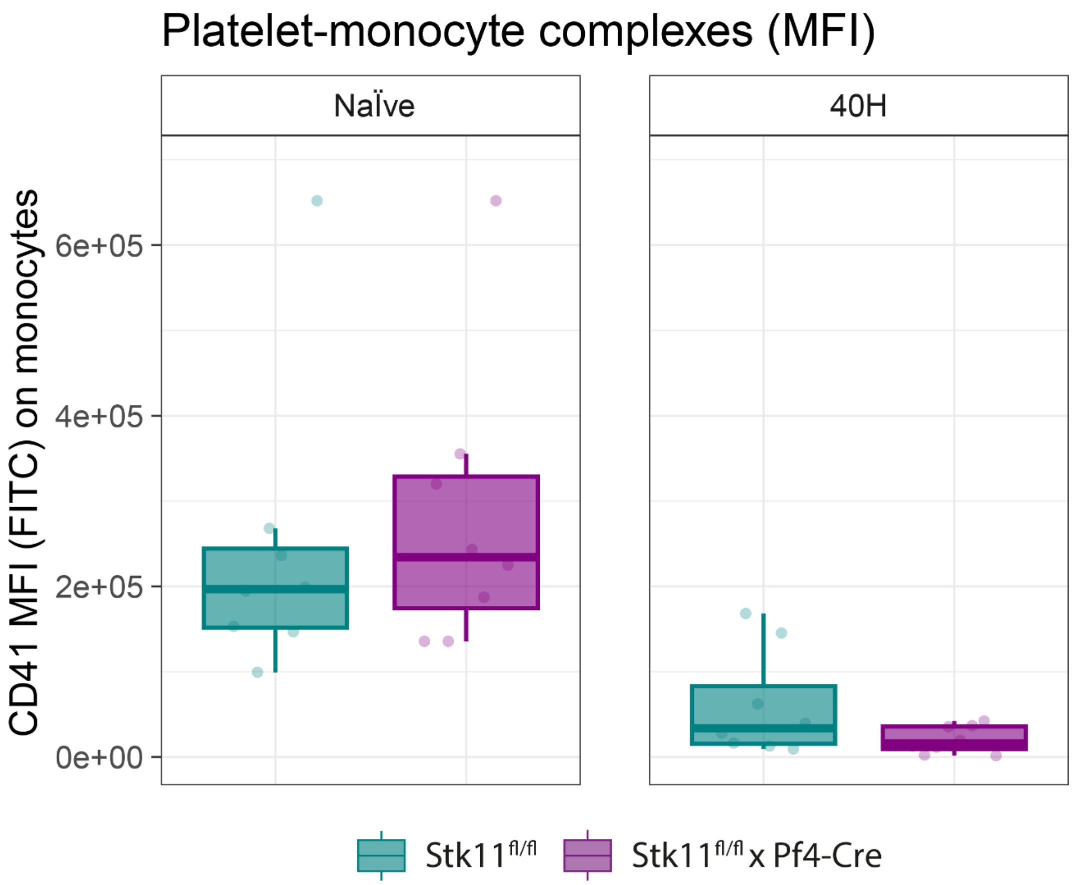

Supplementary Figure S1. Platelet-monocyte complexes. Boxplots comparing the median fluorescence intensity (MFI) for CD41 on monocytes of Stk11/ x Pf4-Cre and Stk11/ littermate control mice (naïve and 40 h after infection). n = 8 per group. *p*-values are derived from BH-adjusted Wilcoxon tests.

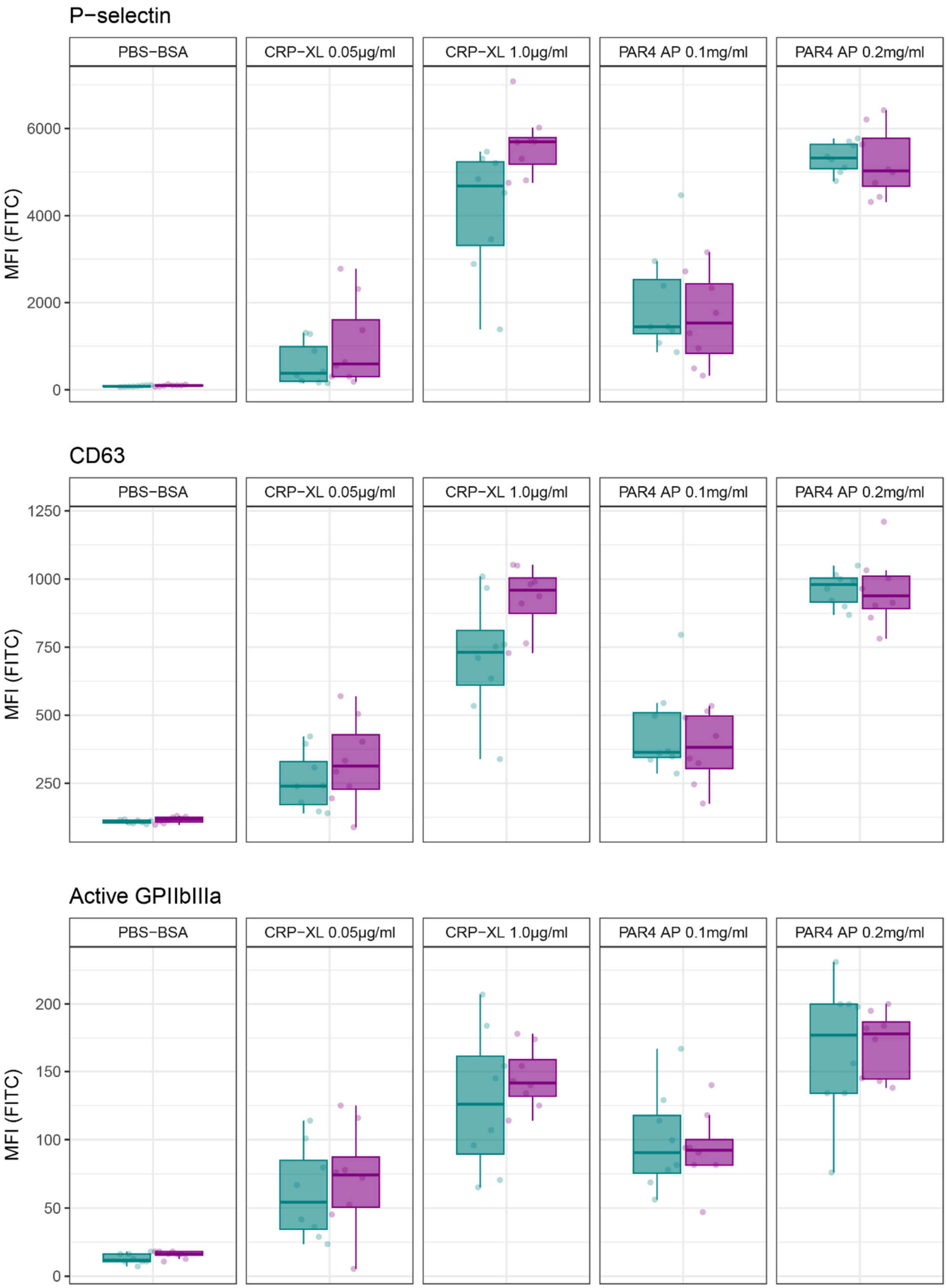

Supplementary Figure S2. Platelet activation 24 h after infection with *K. pneumoniae*. Boxplots comparing the median uorescence intensity (MFI) for P-selectin, CD63 or GPIIbIIIa in active conformation on the platelet surface of Stk11/ x Pf4-Cre and Stk11/ littermate control mice (naïve and 24 h after infection). Each column represents a treatment with a platelet agonist or vehicle (PBS-BSA); n = 8 per group. *p*-values are derived from BH-adjusted Wilcoxon tests.

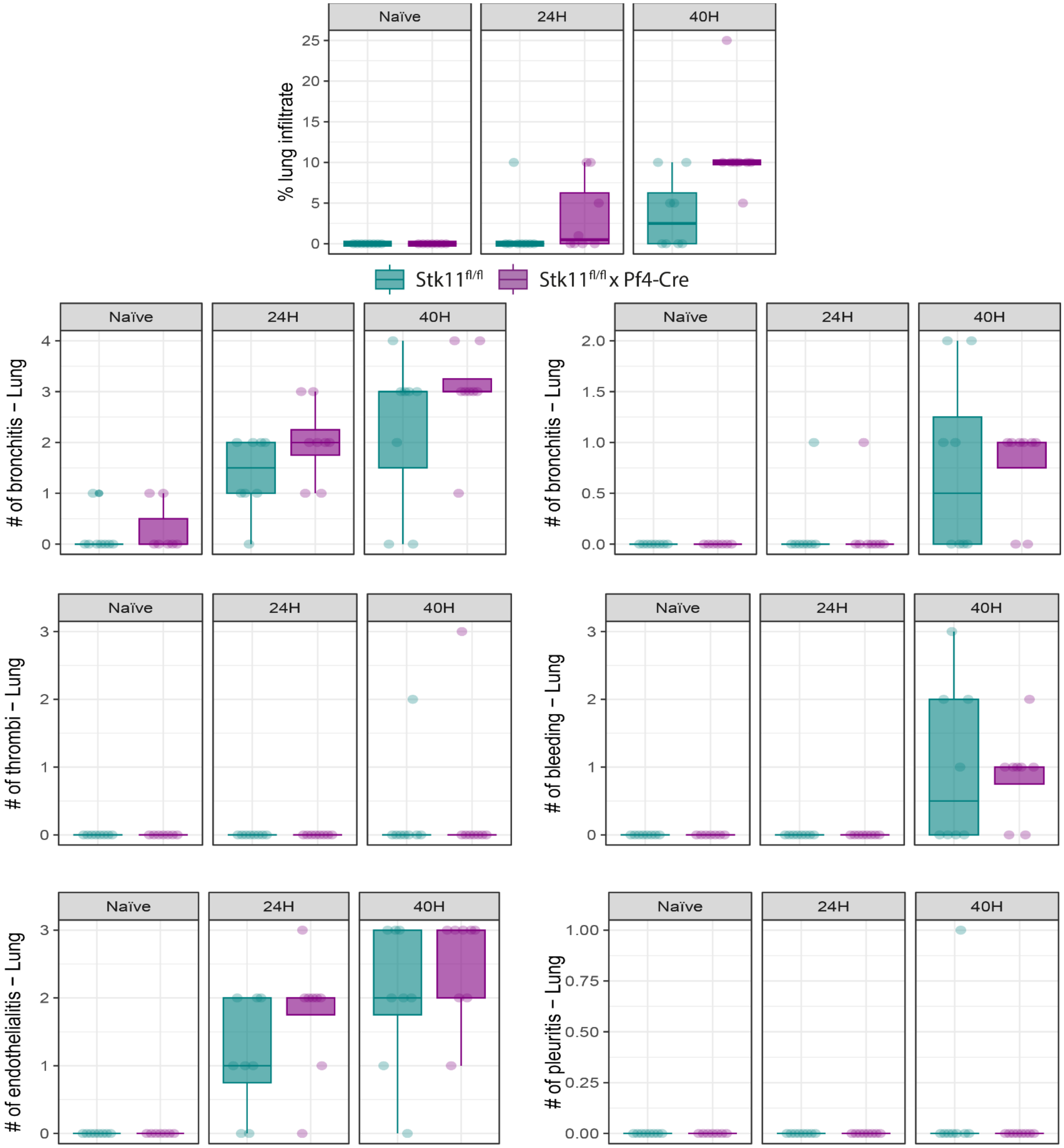

Supplementary Figure S3. Lung pathology scores, stratified by its individual components from H&E stained slides, evaluated by a pathologist blinded to group identity, visualized in m panels: non-infected naïve mice, and in mice 24 or 40 h after *K. pneumoniae* infection.
